# Can MR Enterography and Diffusion-Weighted Imaging Predict Disease Activity Assessed by Simple Endoscopic Score for Crohn’s Disease?

**DOI:** 10.5334/jbsr.1521

**Published:** 2019-01-18

**Authors:** Levent Soydan, Ali Aslan Demir, Serhat Ozer, Selvinaz Ozkara

**Affiliations:** 1 TC Saglik Bakanligi Haydarpasa Numune Egitim ve, Arastirma Hastanesi Istanbul, TR; 2 Istanbul Faculty of Medicine, Istanbul University, TR

**Keywords:** Crohn’s, Disease (CD), MR Enterography, Diffusion, Disease Activity Score

## Abstract

**Purpose:**

Monitoring Crohn’s disease (CD) activity has a crucial importance, especially for evaluating treatment efficacy. Magnetic resonance enterography (MRE) and diffusion-weighted imaging (DWI) or their combination may represent potential non-invasive tools for this purpose. This study aimed to examine DWI and MRE for their potential to differentiate between different grades of ileocolonic CD activity.

**Materials and Methods:**

This retrospective study included 54 adult patients with a diagnosis of CD who underwent ileocolonoscopy and MRE including the DWI sequence. The severity of CD inflammation was categorized by Simple Endoscopic Score for Crohn’s Disease (SES-CD) as inactive, mild, moderate and severe. In addition, following conventional MRE and DWI parameters were examined: bowel wall thickness, mural T2 hyperintensity, contrast enhancement, DWI signal intensity, and apparent diffusion coefficient (ADC) values.

**Results:**

In patients with moderate to severe disease based on SES-CD, T2 hyperintensity score [1.68 ± 0.77 (1–3) vs. 2.19 ± 0.69 (1–3); p = 0.013] and mean DWI score [2.42 ± 0.58 (1–3) vs. 2.04 ± 0.69 (1–3); p = 0.037 ] were higher and mean ADC values [1.5 ± 0.4 (0.9–2.5) vs. 1.2 ± 0.3 (0.6–1.8)] were lower compared to patients with inactive to mild CD. ADC had a moderate diagnostic accuracy in predicting moderate to severe disease (AUC = 0.729, 95% CI = 0.591–0.841, p = 0.001), with a cut-off value of ≤1.47 × 10^–3^ mm^2^/sec yielded 88.5% (23/26) sensitivity, 57.1% (16/28) specificity.

**Conclusion:**

DWI, ADC and T2 signal appear to differentiate moderate to severe CD from inactive to mildly active CD, based on SES-CD evaluation and may be useful in monitoring disease activity, particularly when evaluating treatment response.

## Introduction

Crohn’s disease (CD) is a chronic disorder of the gastrointestinal tract that may affect any segment, with a predilection for the terminal ileum. It is characterized by the segmental enteric and transmural inflammation of the bowel wall, leading to erosions and ulcerations that ultimately result in the formation of sinuses, fistulae and abscesses as well as inflammatory strictures when untreated [1,2]. The treatment aims at both achieving mucosal healing confirmed by endoscopy in the short-term, and controlling disease severity (i.e., disease activity) in the long-term [3]. Therefore, monitoring disease activity during disease course has a crucial importance for evaluating treatment efficacy.

Although there is no consensus regarding a single standard of reference, colonoscopy has been conventionally used for the assessment of disease activity [4]. However, it is invasive, requiring anesthesia and absence of bowel stenosis. In addition, complications of CD may limit its repetitive use during the course of the disease [3,4]. Lastly, the terminal ileum may not be accessed in all cases [5,6]. In order to overcome these challenges, various indices incorporating clinical and endoscopic parameters have been developed to estimate disease activity. Simple Endoscopic Score for Crohn’s Disease (SES-CD) uses the virtue of endoscopy to directly visualize and evaluate the mucosal surface, and has been developed in an attempt to provide simple, reproducible, and easily applicable index with high accuracy [7]. Although SES-CD has limitations such as the lack of consensus on its timing and severity grading, it appears to be more reliable and more responsive to changes in CD activity as compared to other endoscopic indices [8,9].

Magnetic resonance enterography (MRE) is usually not considered as a first line method but it may provide information about CD activity with relatively high accuracy and reproducibility [4,5,10]. Findings of several studies indicate that MRE may predict disease activity [1,11,12,13,14,15]. However, contrast material needs to be administered for MRE, and its accuracy still needs to be improved. Diffusion-weighted imaging (DWI), on the other hand, does not need contrast administration and is being increasingly added to MRE to improve diagnostic accuracy [16]. DWI is based on the movement of random water molecules in the bowel wall at the micromolecular level [2]. Inflammation of bowel wall leads to reduced movement of water molecules thereby eliciting increased diffusion signal along inflamed bowel segments [3]. Although negatively affected by bowel motion and T2 shine-through effect, several studies reported high accuracy rates for DWI in detecting inflamed intestinal segments as well as in distinguishing active from inactive disease [4,17,18,19,20]. The accuracy of DWI to differentiate between different grades of CD inflammation has also been investigated against endoscopic references with promising results [21,22,23]. Thus, MRE and DWI or a combination thereof may represent potential non-invasive tools for monitoring CD activity.

The aim of this study was to examine the abilities of DWI and MRE in differentiating different grades of ileocolic CD activity as defined by SES-CD.

## Methods

### Study population and design

This retrospective study included 54 adult patients with a histological diagnosis of CD who presented with abdominal complaints between March 2015–March 2016 and underwent ileocolonoscopy and MRE including DWI. The mean duration between ileocolonoscopy and MRE was 18.0 ± 19.7 days, during which no medication was given. Patients with a history of bowel surgery for CD and emergency cases were excluded. The study was approved by local ethics committee and conducted in accordance with the latest version of Helsinki Declaration.

### Ileocolonoscopic evaluation

**Figure 1:**
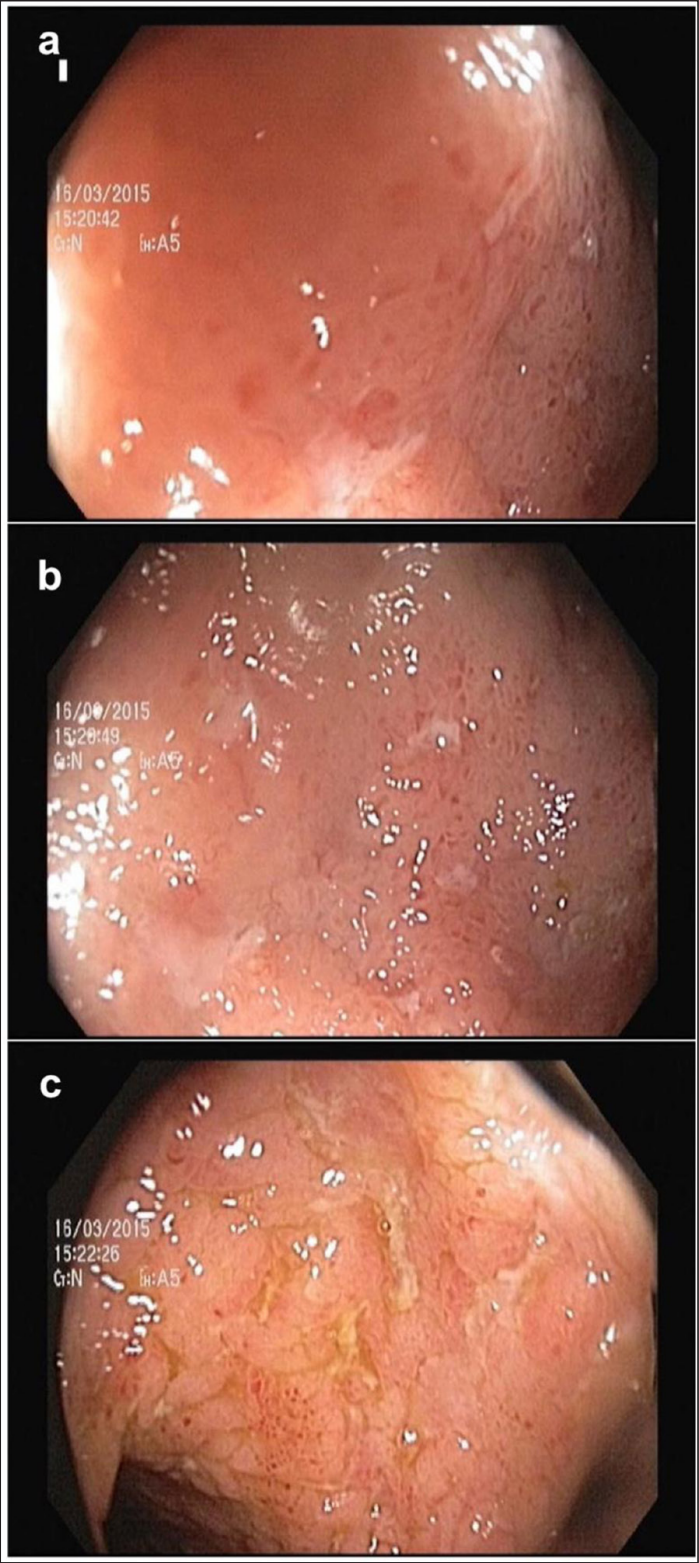
Endoscopic appearances of terminal ileitis with aphthous lesions **(a, b)** and linear ulcers **(c)**.

Ileocolonoscopy was performed by two gastroenterologists using endoscopy devices of Olympus evis exera II (Olympus, Japan) and Fujinon VP-4450 HD (Fujinon, Japan). All colonic segments could be passed up to the terminal ileum and visualized adequately (Figure 1). The entire ileocolonic tract was subdivided into five segments: terminal ileum (distal 15–20 cm of ileum), right colon (cecum and ascending colon), transverse colon, left colon (descending colon and sigmoid), and rectum. The severity of CD inflammation in each segment was scored between 0 and 3 by SES-CD as follows: presence and size of ulcers on the mucosal surface, affected ulcerated area, proportion of affected surface, and presence or absence of narrowing [7]. A segmental SES-CD score ranging between 0–12 was calculated by summing these scores. Finally, a total SES-CD for each patient was calculated from the sum of the segmental scores. Overall disease activity was then inferred from the total SES-CD for each patient based on the following classification: 0–2 inactive, 3–6 mild, 7–15 moderate, >15 severe [24]. Thus, patients with a SES-CD score ≥ 7 were categorized having moderate to severe disease.

### Conventional MRE and DWI procedures

Forty-five patients received low-residue diet for five days before MRE, fasted overnight, and took oral laxatives for bowel cleansing; 9patients fasted at least six hours before the procedure. On the day of examination, patients were given 1200–1500 ml of 3% mannitol solution over 50 min to create a biphasic contrast effect throughout the examined segments. To reduce peristaltic activity 0.5 mg glucagon was administered intramuscularly 30 min before imaging. MRE was then performed using 1.5 T General Electric optima 450 w MR unit (GE Healthcare, Milwaukee, Wisconsin, USA) with multi-channel abdominal phase array coils. LAVA (Liver acquisition with volume acquisition) sequences were used to acquire post-contrast images using 0.1 mmol/kg of gadolinium chelate (Omniscan®, Nycomed Imaging, Oslo, Norway) which was administered intravenously following a delay of 70 seconds. No late phase was performed. Details of MRE scan parameters are shown in Table 1.

**Table 1:** MR enterography protocol.

Sequence	TR/TE (msec)	ST/gap (mm)	Matrix	SENSE factor	FOV (cm)	NEX	FA

**SSFSE (T_2_W)**	710–800/130	6/0	320 × 224	2	47	1	NA
**2D FIESTA (fat-sat)**	4/2	6/0	288 × 256	2	42	1	75°
**DWI (b:0, 800 sec/mm^2^)**	6000/70	6/1	160 × 224	NA	46	4	90°
LAVA (fat-sat T_1_W)*	6.1–6.4/1.9	4/1	320 × 192	NA	40	1	12°

Abbreviations: MR, magnetic resonance; ST, slice thickness; SENSE, sensitivity encoding; TR/TE, repetition time/echo time; FOV, field of view; NEX, number of excitations; FA, flip angle; SSFSE, single shot fast spin echo; T_2_W, T_2_-weighted; fat-sat, fat saturated; 2D FIESTA, two-dimensional fast imaging employing steady-state acquisition; DWI, diffusion-weighted imaging; LAVA, liver acquisition with volume acquisition, NA, not applicable. * before and after gadolinium contrast administration. Total scan duration: 25 minutes; frequency encoding direction: right to left; acquisition planes are coronal and axial in all sequences; all sequences were performed with respiratory triggering except for the T1W imaging which was obtained breath-held.

MRE assessment was performed by the consensus of two radiologists (LS and AAD) who were blinded to SES-CD scores. DWI signal scoring preceded conventional MRE image reading to ensure blinding. Image analyses were conducted on a post-processing workstation (Advantage Windows version AW 4.6 Functool software AW 4.6, General Electric Medical Systems, Milwaukee, WI, USA). On MRE images ileum, right colon, transverse colon, left colon, and sigmoid were identified and evaluated separately, however radiological measurements were made only from the segment with most marked MRE abnormalities including marked mural contrast enhancement on postcontrast T1 sequences and wall thickening/irregularities on T2 sequences and most notable DWI signal hyperintensity.

The following conventional MRE parameters were analyzed and semiquantitative scoring was assigned to each parameter as follows: bowel wall thickness defined as mild (0–4 mm = 1), moderate (5–7 mm = 2), marked (>7 mm = 3); mural T2 hyperintensity (or mural edema) defined as hyperintensity of ileum or colon wall relative to psoas muscle signal which was classified as mild (dark grey = 1), moderate (light grey = 2) and marked (grey-white intensity = 3); contrast enhancement along the affected segment defined as mild (mucosal enhancement only = 1), moderate (all bowel wall enhancing equally, i.e. transmural = 2), marked (transmural and serosal enhancement with central band of relatively reduced enhancement = 3).

**Figure 2:**
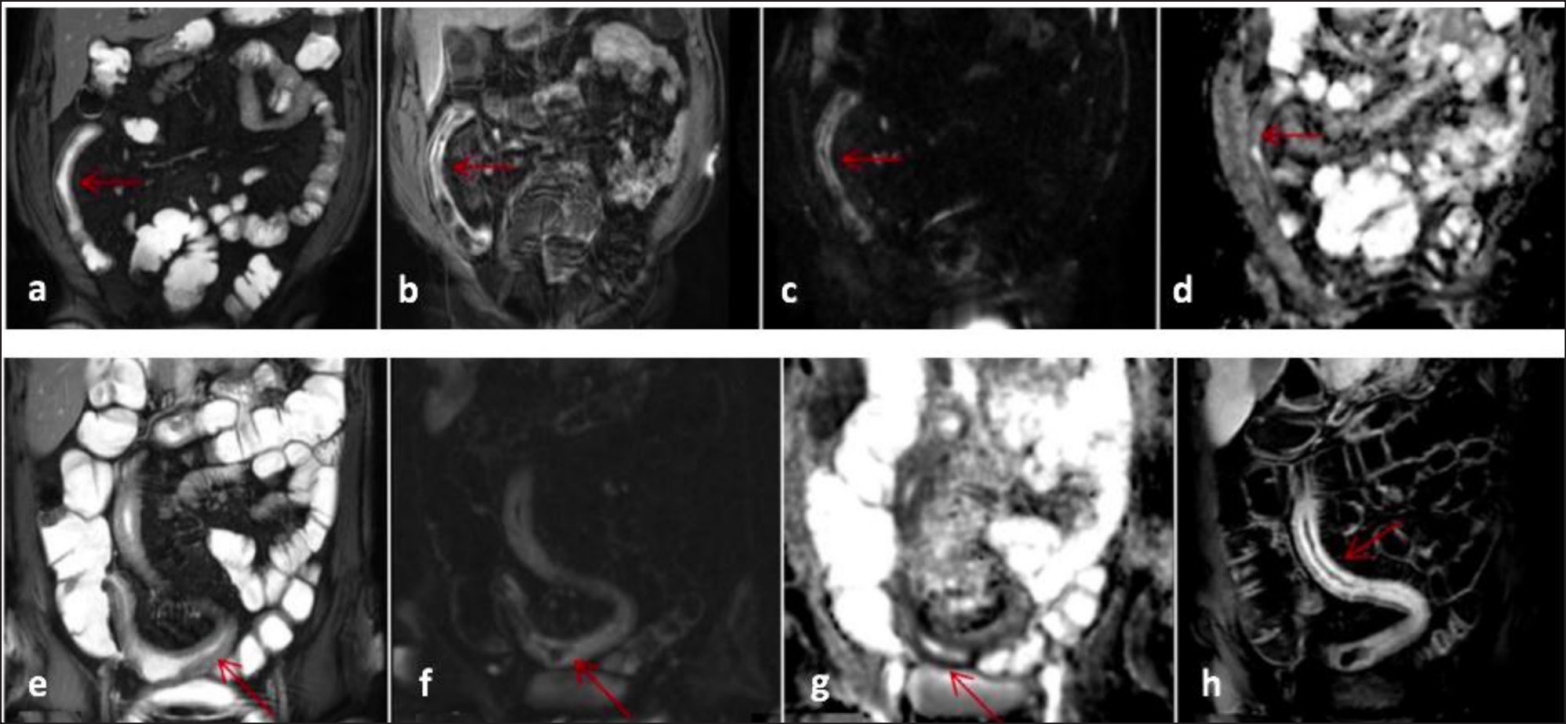
MR images of a patient with Crohn’s disease involving distal ileum **(a–e)** and another patient with involvement of a long segment of distal ileum and ileocecal junction **(e–h)**. The images of the first patient show wall thickening and mural T2 hyperintensity on coronal FIESTA image (a), contrast enhancement along thickened ileal wall (b), mural diffusion restriction as suggested by DWI hyperintensity (c) with corresponding signal decrease along thickened ileal wall on ADC mapping (d). The images of the second patient show diffuse mural T2 signal increase with ileal wall thickening on coronal FIESTA image (e), mural DWI hyperintensity along involved ileal segment (f) with corresponding mural hypointensity on ADC mapping consistent with restricted diffusion (g) and mural contrast enhancement after intravenous gadolinium (h).

A semiqualitative scoring was also obtained for the DWI signal as follows: 1, mild (≤renal cortex); 2, moderate (>renal cortex and <spleen); or 3, marked (≥spleen). The ADC map that was generated from DWI images using b values 0 s/mm^2^ and 800 s/mm^2^ was used to measure ADC values from the segments with notable DWI hyperintensity and morphological abnormalities such as marked mural contrast enhancement on T1 weighted sequences and wall thickening and irregularity on T2 sequences (Figure 2). On magnified images, each of the two radiologists measured mean ADC values by placing three round regions of interest (ROI) on areas of affected bowel wall with most prominent abnormalities and calculated a mean value for that segment. The representative mean ADC for each affected segment was then determined from the mean respective segmental ADC values of each radiologist. The mean area of the ROI was 30.0 ± 2.5 mm^2^.

### Statistical analysis

The statistical analysis was performed by using the Number Cruncher Statistical System software (NCSS 2007, Kaysville, Utah, USA). The difference of quantitative data between two groups was evaluated by Student t-test or Mann Whitney U-test for normally and non-normally distributed variables, respectively. For the comparison of three and more groups, analysis of variance (ANOVA) or Kruskal-Wallis test was used, based on normality of data, where Tukey test was used for pairwise comparisons. For comparison of qualitative data, Pearson chi-square and Fisher-Freeman-Halton tests were used. Receiver operator characteristic curves (ROC) were generated to examine the accuracy of estimations and potential cut-off values. The accuracy of parameters to predict the outcome was given as area under the curve (AUC) with 95% confidence interval (CI). The cut-off value of ADC to predict CD activity was evaluated for its diagnostic parameters. In addition, these parameters were calculated for relevant dichotomous variables. For inter-observer agreement of MRE and DWI parameters, the kappa values and intra-class correlation coefficient were calculated. A p value smaller than 0.05 was considered statistically significant.

## Results

### Clinical characteristics of patients

Of 54 patients, 20 (37.0%) were follow-up patients and 34 (63.0%) were newly diagnosed CD. Because MRE assessments were made on a one-segment-per-patient basis, a total of 54 segments of 54 patients were evaluated. Details of clinical characteristics are shown in Table 2.

**Table 2:** Demographic and clinical characteristics of the study patients.

Characteristic	n = 54

Female gender	29 (53.7%)
Age, years (mean ± SD)	39.9 ± 14.2
Length of involved segments, cm (mean ± SD)	14.7 ± 11.2
Duration of the disease, years (mean ± SD)	4.6 ± 4.9
Time between MRE and colonoscopy, days (mean ± SD)	18.0 ± 19.7
Location of Crohn’s disease	
Ileum	12 (22.2%)
Colon	10 (18.5%)
Ileum and colon	32 (59.3%)
Behavior of Crohn’s disease	
Non-stricturing, non-penetrating	26 (48.1%)
Stricturing	4 (7.4%)
Penetrating	24 (44.4%)
CDAI, (mean ± SD)	224.4 ± 122.1 (18–548)
Receiving any medication for CD*	36 (66.6%)
CRP, mg/dl (mean ± SD)	2.31 ± 2.89

Unless otherwise stated, data presented as number (percentage). * Receiving one of the following medications or a combination: azathioprine, 5-aminosalicylic acid, biological therapy, or steroids. The normal limit of CRP in our laboratory was <5 mg/L. CDAI, Crohn’s Disease Activity Index; CRP, C-reactive protein; CD, Crohn’s disease; SD, standard deviation; MRE, magnetic resonance enterography.

### MRE and DWI parameters with respect to SES-CD

The mean SES-CD score of the patients was 7.4 ± 5.1 (range 0–21). According to the SES-CD, 8 patients had inactive CD, 20 patients had mildly active CD, and 26 patients had moderate/severe CD. Table 3 compares patients with inactive/mild versus moderate/severe disease in terms of MRE and DWI parameters.

**Table 3:** Comparison of patients with inactive/mild versus moderate/severe disease based on SES-CD in terms of conventional MRE and DWI parameters.

	SES-CD < 7 (Inactive/mild CD) n = 28	SES-CD > 7 (Moderate/severe CD) n = 26	p

*Conventional MRE parameters*			

Wall thickness			
Mild (0–4 mm)	6 (21.4%)	8 (30.8%)	0.545
Moderate (5–7 mm)	9 (32.2%)	10 (38.4%)	
Marked (>7 mm)	13 (46.4%)	8 (30.8%)	
Mean mural T2 hyperintensity (score)	1.68 ± 0.77 (1–3)	2.19 ± 0.69 (1–3)	0.013
Mild (dark grey)	14 (50.0%)	4 (15.4%)	
Moderate (light grey)	9 (32.1%)	13 (50.0%)	
Marked (grey-white)	5 (17.9%)	9 (34.6%)	
Contrast enhancement			
Mild (mucosal)	1 (3.6%)	1 (3.8%)	0.789
Moderate (transmural)	13 (46.4%)	10 (38.5%)	
Marked (transmural and serosal)	14 (50.0%)	15 (57.7%)	
Mean *DWI score*	2.42 ± 0.58 (1–3)	2.04 ± 0.69 (1–3)	0.037
Mild	6 (21.4%)	1 (3.8%)	
Moderate	15 (53.6%)	13 (50.0%)	
Marked	7 (25.0%)	12 (46.2%)	
DWI plus T2 score > 3	13 (46.4%)	13 (88.5%)	0.001
DWI plus T2 score > 4	6 (21.4%)	14 (53.8%)	0.014
ADC	1.5 ± 0.4 (0.9–2.5)	1.2 ± 0.3 (0.6–1.8)	0.003

SES-CD, Simple Endoscopic Score for Crohn’s Disease; MRE, magnetic resonance enterography, DWI, diffusion-weighted imaging; ADC, apparent diffusion coefficient.

When three groups of patients with inactive, mildly active and moderate to severe CD were compared with respect to ADC values, a significant difference was found (p = 0.012). Mean ADC in moderate to severe CD group was significantly lower than the mildly active disease group (1.2 ± 0.3 vs. 1.5 ± 0.4, p = 0.028). However, no statistically significant difference was found between the ADC values of the inactive versus mild group (1.5 ± 0.3 vs. 1.5 ± 0.4, p = 1.000) or moderate/severe group (1.5 ± 0.3 vs. 1.2 ± 0.3, p = 0.069). Inactive, mild and moderate/severe groups did not differ with regard to the distribution of T2 hyperintensity, DWI signal, wall thickness, and contrast enhancement scores (p = 0.087, 0.188, 0.141, and 0.931, respectively).

When inactive and mild groups were combined, lower T2 hyperintensity was less common (p = 0.013), DWI score was higher (p = 0.037), and ADC values were lower (p = 0.003) in moderate/severe CD group than in inactive/mild CD group (Table 3). In addition, a high combined DWI plus T2 score was more common among patients with moderate to severe disease (p = 0.001 and 0.014 for a combined score > 3 and > 4, respectively). Groups did not differ with regard to wall thickness and contrast enhancement distribution (p > 0.05 for both).

### Accuracy of ADC values in predicting moderate/severe CD activity

**Figure 3:**
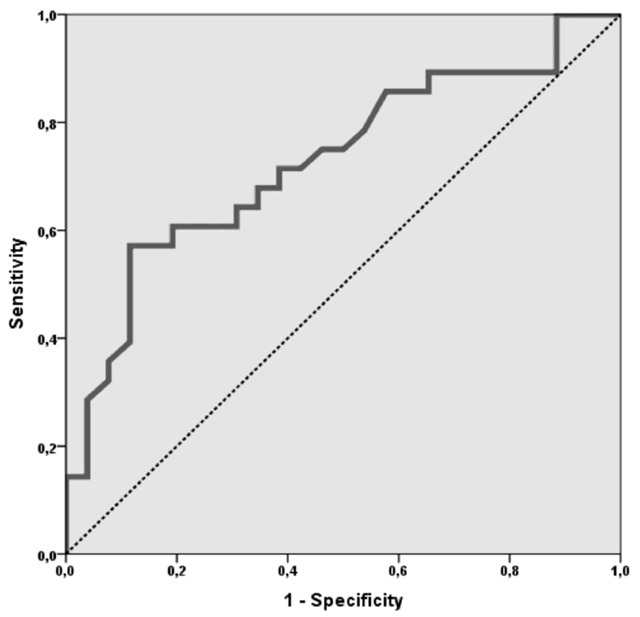
ROC curve of ADC for diagnosing SES-CD-based moderate to severe CD activity.

Figure 3 shows the ROC curve of ADC for diagnosing SES-CD-based moderate to severe CD activity. ADC values has moderate diagnostic accuracy (AUC = 0.729, 95% CI = 0.591–0.841, p = 0.001). A cut-off ADC value of ≤ 1.47 × 10^–3^ mm^2^/sec yielded 88.5% (23/26) sensitivity, 57.1% (16/28) specificity, 65.7% (23/35) PPV and 84.2% (16/19) NPV for predicting moderate to severe CD activity. Table 4 shows diagnostic performance parameters for different MRE and DWI variables. A combination score (DWI plus T2) revealed a good sensitivity 88.5% (23/26) and a 53.6% (15/28) specificity.

**Table 4:** Diagnostic performance parameters for MRE and DWI in predicting SES-CD based moderate to severe disease.

	Sensitivity (%)	Specificity (%)	PPV (%)	NPV (%)	Diagnostic Accuracy (%)

ADC < 1.47 × 10^–3^ mm^2^/sec	88.5 (23/26)	57.1 (16/28)	65.7 (23/35)	84.2 (16/19)	72.22 (39/54)
T2 hyperintensity score > 1	84.62 (22/26)	50.00 (14/28)	61.11 (22/36)	77.78 (14/18)	66.67 (26/54)
T2 hyperintensity score > 2	34.62 (9/26)	82.14 (23/28)	64.29 (9/14)	57.50 (23/40)	59.26 (32/54)
DWI score > 1	96.15 (25/26)	21.43 (6/28)	53.19 (25/47)	85.71 (6/7)	57.41 (31/54)
DWI score > 2	46.15 (12/26)	75.00 (21/28)	63.16 (12/19)	60.00 (21/35)	61.11 (33/54)
DWI plus T2 score > 3	88.46 (23/26)	53.57 (15/28)	63.89 (13/36)	83.33 (15/18)	70.37 (38/54)
DWI plus T2 score > 4	53.85 (14/26)	78.57 (22/28)	70.00 (14/20)	64.71 (22/34)	66.67 (36/54)

SES-CD, Simple Endoscopic Score for Crohn’s Disease; MRE, magnetic resonance enterography, DWI, diffusion-weighted imaging; ADC, apparent diffusion coefficient; PPV, positive predictive value; NPV, negative predictive value.

### Inter-observer agreement

The kappa values of inter-observer agreement were 0.79 for DWI score, 0.81 for contrast enhancement, 0.90 for wall thickness, and 0.67 for T2 hyperintensity; the intra-class correlation coefficient was 0.92 for ADC values (all p-values < 0.001).

## Discussion

This study found that DWI findings and T2 signal may be helpful in differentiating moderate to severe CD activity from less severe forms of the disease, which may be particularly helpful for disease monitoring. This study is among few studies investigating the diagnostic performance of individual or combined MRE/DWI parameters in discriminating between disease activity levels as assessed by SES-CD.

Previous studies indicated that mural T2 hyperintensity and bowel wall thickness were independent predictors of CD activity assessed by hispathological evaluation [1,18,25]. Higher mural T2 signal, probably reflecting mural edema, was found to be associated with higher degrees of inflammation [18,25,26]. Similarly, this study found a significant association with T2 signal hyperintensity and disease activity, particularly when this parameter is combined with DWI score. The use of contrast enhancement and wall thickness measurements have been studied in MRE with controversial results [1,11,18,22,25]. It has been suggested that the measurement of contrast enhancement may be non-reproducible depending on dynamic imaging protocols and thus may vary among researchers [3,19]. The lack of rectal preparation before the MRE study and the presence of undistended intestinal and colonic segments in some patients may both lead to false positive wall thickening [11,22,27]. In this study, neither contrast enhancement nor wall thickness showed significant association with disease severity.

The efficacy of DWI in CD has also been investigated before, although less than conventional MRE. Studies reported that DWI can distinguish inflamed segments from normal segments with similar diagnostic accuracies as conventional MRE [2,17,23]. A meta-analysis yielded 92.9% sensitivity and 91% specificity for DWI in diagnosing active bowel wall inflammation, which was higher than contrast-enhanced MRE [20]. However, high diagnostic accuracies were more common in studies with no blinding of DWI to MRE findings and in studies which used contrast-enhanced MRE as reference standard instead of external references like endoscopy or histopathology. Because of these and the large heterogeneity between these studies, the apparent high diagnostic accuracies of DWI were likely overestimations [4,20]. On the other hand, this study showed significant associations between SES-CD based disease activity versus ADC and DWI signal despite using an external reference standard (ileocolonoscopy) and DWI readings blinded to MRE, both of which may be regarded as strengths of the study.

It has been suggested that the addition of DWI to conventional MRE would lead to higher diagnostic yields, though only with a marginal benefit [17,20,21]. However, some controversy existed whether DWI could replace conventional MRE [4,17,19,20,28]. For DWI to completely obviate and replace MRE, it has been suggested that DWI should reveal inflammation unidentified by MRE [20]. More controversy exists about ADC, the objective quantitative measure of DWI, in both the diagnosis and the severity grading of CD [3,4,6,17,18,21,23]. In the present study, we investigated whether MRE and DWI signal can differentiate between inactive, mild and moderate-severe active CD, as defined by SES-CD and found only significant associations for moderate to severe disease activity. Lower ADC and higher DWI signal was more common in higher degrees of bowel inflammation, which was in agreement with previous studies [2,17,20,23,29]. Increased tissue cellularity, viscosity, dilated lymphatics and granuloma formation has been proposed as possible factors to account for the increased restricted diffusion in inflamed segments [21,23]. In addition, this study revealed that the combined use of DWI signal and T2 signal yielded higher diagnostic accuracy than the use of DWI and T2 signal alone in differentiating higher degrees of CD activity (moderate-severe) from less active CD. We found that DWI and T2 scores > 3 were associated with highest diagnostic accuracy, sensitivity and specificity (70%, 88.5%, 53.6%, respectively); see Table 4.

It is to be noted that we found ADC to perform slightly better than DWI score and MRE parameters in differentiating moderate/severe CD from inactive/mild CD. This suggests that ADC is a more useful tool than conventional MRE in distinguishing between different grades of CD activity although in our study ADC and MRE parameters could not significantly distinguish inactive CD from mildly active CD. We analyzed only the most prominent segment in our study, which may account for this lack of differentiation between all grades. Indeed, other studies conducted with higher numbers of analyzed segments per patient reported that ADC and DWI can distinguish between mildly active CD and inactive CD with high accuracy [23]. Various ADC cut-off values have been suggested in studies with different accuracies [23,30]. Ninivaggi et al. reported that an ADC cut-off of 2.416 × 10^–3^ mm^2^/s yielded 100% sensitivity and specificity to discriminate normal from inflamed bowel segments [31]. Yet no definitive threshold value for ADC has been established which can accurately differentiate active from inactive CD probably because of different sample sizes and different b-values used in the studies [21]. In our study we found that an ADC cut-off of 1.47 × 10^–3^ mm^2^/s yielded a moderate diagnostic accuracy (72%). However, establishing a precise cut-off value may be challenging due to following reasons: (i) poor reproducibility among different scanners [4,6,17,29,32], (ii) complexity of ADC measurement from thin walls, particularly in the presence of peristaltic artifacts [4,17,18], and (iii) overestimation of ADC in normally thin bowel walls or in walls thinned by fibrosis. In addition, selection of higher b values minimizes the perfusion effect of DWI, which should also be considered in interpreting ADC values [17,32,33].

Our study has several limitations. Firstly, since we took measurements from a single segment in each patient, the total number of examined segments was small resulting in smaller numbers within each subgroup, which might have led to diminished diagnostic accuracy in differentiating between inactive and mildly active CD. Secondly, we used a semiquantitative scoring in the evaluation of images, which may be less accurate than a quantitative scoring and may limit generalizability, although our interobserver agreement was good. Thirdly, our study cohort was heterogenous consisting of patients who were on different therapeutic regimens and of patients who did not receive any treatment at all at the time of examinations. Finally, we only focused on the intestinal and colonic findings of CD and did not evaluate the extraintestinal manifestations of the disease.

## Conclusion

Implementation of DWI, ADC and T2 signal appears to differentiate moderate to severe CD from mildly active to inactive CD, but not inactive CD from mildly active CD. Given the challenges in standardizing ADC values and promising findings of our study supporting the benefits of combining DWI and T2 hyperintensity scores, we suggest the use of this combination rather than relying on ADC alone. Thus, combined use of DWI and T2 signal measurements seems to aid monitoring disease activity, especially in small bowel CD with well distension, obviating the need for contrast administration during MRE and reducing the need for repetitive colonoscopies. Nevertheless, studies with larger numbers of patients and higher number of bowel segments examined should be performed to confirm the diagnostic value of these parameters. Finally, the cost-effectiveness of MRE should be assessed in further studies with larger populations as these examinations will likely be needed repetitively during the course of CD [10].
